# The use of three murine genetic models of Alzheimer’s disease reveals efficacy of 4′,5,7-trihydroxyisoflavone in alleviating cognitive deficits and reducing levels of amyloid-beta and hyperphosphorylated tau protein

**DOI:** 10.1186/s42826-026-00302-9

**Published:** 2026-10-22

**Authors:** Lidia Gaffke, Aneta Szulc, Magdalena Podlacha, Dorota Myślińska, Karolina Pierzynowska, Grzegorz Węgrzyn

**Affiliations:** 1 Department of Molecular Biology, Faculty of Biology, University of Gdańsk, Wita Stwosza 59, Gdańsk, 80-308 Poland; 2 Department of Animal and Human Physiology, Faculty of Biology, University of Gdańsk, Wita Stwosza 59, Gdańsk, 80-308 Poland

**Keywords:** Genistein, Murine models of Alzheimer’s disease, 5xFAD mice, THY-Tau22 mice, 3xTg-AD mice, Improvement of cognitive functions, Beta-amyloid and p-tau reduction

## Abstract

**Background:**

Despite intensive studies on developing therapies for Alzheimer’s disease (AD), there is still a need to improve the treatment efficacy. Results of recent studies indicated that genistein (4′,5,7-trihydroxyisoflavone) was effective in an autophagy stimulation-dependent improving of cognitive deficits and reducing levels of amyloid-beta and hyperphosphorylated tau protein in brains, when employing a streptozotocin-induced rat model of the sporadic form of AD. Despite those encouraging results, the above-mentioned model has its limitations, mainly related to the rapid and inducible character of the symptoms caused by streptozotocin, induction of other effects (like diabetes) by this toxin, and the requirement for invasive administration of this compound to the brain, in contrast to naturally occurring AD. Thus, in this study, we hypothesized that genistein might be effective in reducing symptoms of AD in well-defined genetic models of this disease and tested the efficacy of this compound.

**Results:**

Three genetic models of AD were employed, including mouse lines 5xFAD, THY-Tau22, and 3xTg-AD. Orally administered genistein (at the dose of 150 mg/kg/day) considerably alleviated cognitive deficits (in spatial memory, learning, and long-term memory) of AD animals as estimated using the Morris water maze and the novel object recognition tests. Moreover, significantly reduced levels of amyloid-beta and hyperphosphorylated tau protein in plasma and brains were detected in genistein-treated AD animals relative to those treated with water.

**Conclusions:**

We conclude that genistein revealed significant efficacy in ameliorating symptoms occurring in murine genetic models of AD. Thus, further studies towards the development of a genistein-based treatment for this disease are substantiated.

**Clinical trial number:**

Not applicable.

**Supplementary Information:**

The online version contains supplementary material available at https://doi.org/10.1186/s42826-026-00302-9.

## Background

Alzheimer disease (AD) is the most common neurodegenerative disorder [1]. The major symptoms include memory impairment, cognitive deficit, difficulty in decision-making, and behavioral changes. There are two forms of AD, familial and sporadic. The familial form occurs in 1–5% of patients and is caused by specific variants of different genes, including *APP* (coding for amyloid-β precursor), *APOE* (encoding apolipoprotein E), *CLU* (encoding clusterin), *PSEN1* and *PSEN2* (encoding presenilin 1 and 2, respectively), and others [2, 3]. The presence of pathogenic variants in the *APP*, *PSEN1*, and *PSEN2* genes is recognized as causal for familial AD (essentially deterministic rather than probabilistic), whereas mutations in other genes, such as *APOE* and *CLU*, are generally considered risk factors for sporadic AD [2]. The sporadic form occurs in 95% or more patients and is a multifactorial disease whose etiology is not yet completely understood [4]. Both forms are characterized by amyloid plaques and intraneuronal neurofibrillary tangles, occurring mostly in the central nervous system [5]. These pathological structures consist of amyloid-β (Aβ) and hyperphosphorylated tau protein (p-tau), respectively.

Although studies on AD have been extensive for decades, no effective treatment is available to date. The statement remains valid irrespective of the current availability of formally registered anti-AD drugs, based on anti-Aβ antibodies, Aducanumab and Lecanemab [6, 7]. This is because the clinical effects of these therapeutics are not fully satisfactory, despite patients showing lower Aβ levels [8, 9]. It is also worth mentioning that various potential drugs that were effective in cellular and animal models failed to improve clinical symptoms in humans [6]. This was possibly because those approaches targeted Aβ accumulation as the only cause of AD in the models; thus, treatments focused solely on reducing Aβ levels were effective. However, data from human studies suggest that the sporadic AD form is caused not only by Aβ but also by p-tau and other pathological factors, similarly to the significantly more complicated pathomechanisms of familial AD than just accumulation of Aβ. Moreover, complex neurodegenerative changes in the brains of AD patients were described, showing multi-factorial neurodegeneration as the central component of the disease process, and production of Aβ by non-neural cells as an important component of the disease [5]. Therefore, eliminating of just one factor, such as Aβ, and considering only neurons, are inefficient, and new anti-AD therapies are urgently needed.

One might propose that to solve the problem of AD, as a currently incurable and unpreventable disease, is to find a way to eliminate all (not just one) of its primary causes. Since the crucial molecules that cause neurodegeneration in AD are improperly folded proteins (Aβ and p-tau) and many other aggregate-forming macromolecules, exemplified by differently modified heparan sulfate [8], it appears reasonable to seek a method capable of removing all these improper structures. In this light, stimulation of autophagy, a natural process that can decay improper macromolecules, might allow for the clearance of the toxic aggregates. However, while the potential role of autophagy in treating neurodegenerative diseases was suggested [10, 11], studies systematically assessing the question of whether a single compound could be used to reduce levels of Aβ, p-tau and possibly other aggregates, and thus, if it could be a therapeutic strategy for AD, are scarce. The main problem is that overstimulating autophagy might lead to auto-destruction of cells; hence, the challenge is to find an autophagy stimulator that fulfills the specific requirements for use as an efficient AD drug. Such a compound must cross the blood-brain barrier, stimulate autophagy, be non-toxic, and be suitable for long-term administration. All of these features must co-occur in the same agent, a requirement that is not currently met by the vast majority of known autophagy stimulators [12].

Despite the difficulties mentioned above, recent studies suggested that 4’5,7-trihydroxyisoflavone (systematic chemical name: 5,7-dihydroxy-3-(4-hydroxyphenyl)-4-*H*-1-benzopyran-4-one; common name: genistein) may possess all of the features required to be a therapeutic autophagy stimulator potentially useful in AD treatment. Experiments with the streptozotocin-induced rat model of sporadic AD indicated that genistein administration (at the dose of 150 mg/kg/day) for 30–90 days efficiently induced autophagy in the brain, drastically reduced or normalized brain levels of Aβ and p-tau, and corrected behavioural and cognitive parameters of the animals, with no significant adverse effects [13]. Moreover, experiments using a cellular model indicated that the biochemical effects of genistein resulted from autophagy stimulation [13]. Subsequent studies indicated that genistein activates autophagy in a TFEB- and FOXO3-dependent manner [14]. This isoflavone also has other biological activities that might be beneficial in restricting AD symptoms [15–20].

The promising results obtained with the use of genistein in the streptozotocin-induced rat model of AD encouraged us to conduct studies on the efficacy of this compound in more defined animal models of this disease. Apart from the various advantages of the above-mentioned AD model, there are also various limitations, especially the lack of specificity of streptozotocin. Namely, this compound, despite causing AD-like symptoms in rodents when administered by injection into the brain, is also a diabetes-inducing drug and a molecule revealing other toxic activities [21]. Moreover, to induce the symptoms resembling AD, streptozotocin should be administered directly into the brain, through an invasive procedure, which is a significantly different initiation of the disease than the onset of naturally occurring AD [13]. This also implies that effects of this toxin on animals are rapid, in contrast to natural AD, causing considerable problems in assessing the efficacy of tested potential drugs in light of the timing of their administration relative to the disease onset as well as their pharmacodynamics. Hence, here we used three well-defined, murine genetic models of AD to assess the effects of 4’5,7-trihydroxyisoflavone. One of these models is characterized by overproduction of Aβ, the second model synthesizes relatively large amounts of mutant tau protein, which leads to its hyperphosphorylation, and the third model combines features of the first and the second models (see Methods for details). Although a limitation of such models is that they reproduce mechanisms related to familial, rather than sporadic, forms of AD, the use of a set of diverse and complex laboratory animal models could provide a better view of the efficacy of the tested anti-AD compound. Therefore, we assumed that studies using murine genetic models, together with results from previously reported experiments with the sporadic AD animal model [13], should provide comprehensive information about the effects of genistein administration on the reduction of AD symptoms. In this light, we aimed to investigate effects of the tested treatment on (i) memory and learning, and (ii) levels of Aβ and p-tau in plasma and brain tissue. The rationale for such studies arose from the fact that neurocognitive processes are severely affected in both human patients and animal models of AD. At the same time, the aggregate-forming proteins are commonly assessed as the major neurotoxic compounds responsible for AD pathogenesis and the appearance of specific neurodegenerative symptoms. Therefore, the experiments described in this report allowed us to estimate the effects of genistein on AD-related symptoms under controlled in vivo conditions.

## Methods

### Genistein (4′,5,7-trihydroxyisoflavone)

The compound tested in this study as a potential anti-AD drug, genistein (4′,5,7-trihydroxyisoflavone), was purchased from Pharmaceutical Research Institute, Warsaw (Poland) (cat. no. #446-72-0). The purchased chemical was of 99% purity grade. DMSO was used to prepare a stock solution, which was then stored at -20 °C. For preparing the working solutions, the stock solution was diluted in water at least 1000 times; thus, final concentrations of DMSO were 0.1% or lower, which are commonly assessed as being biologically innocuous (placed by the FDA into the safest category of solvents; https://precision.fda.gov/ginas/app/ui/substances/YOW8V9698H, last accessed on 5 July, 2026). The final dose of genistein, used in all experiments, was 150 mg/kg/day. This dose was chosen based on previously reported studies, in which genistein was shown to be effective in autophagy stimulation in the brains of animal models of AD and Huntington’s disease, being able to cross the blood-brain barrier efficiently enough to reveal biochemical activities while causing no significant adverse effects [13, 14].

### Murine models of AD

Three murine genetic models of AD were used: 5xFAD [22], THY-Tau22 [23], and 3xTg-AD [24] (all purchased from Jackson Laboratories, USA; RRID numbers: MMRRC_034840-JAX, MGI:3717232, and MMRRC_034830-JAX, respectively); see Table 1 for characteristics of these models. The C57BL/6J line was used as a wild-type control (purchased from Tri-City Central Animal Laboratory, Research and Service Center of the Medical University of Gdansk, Poland). Males and females of murine AD models and control mice were employed.

**Table 1:** Characteristics of the three murine models of AD used in this study

Model	Genetic modifications and gene variant(s)	Biochemical and anatomical changes and onset of appearance	Main symptoms and onset of appearance	Reference(s)
5xFAD	Overexpression of human *APP* gene (with K670N/M671L, I716V, and V717I variants) and *PSEN1* (with M146L and L286V variants), both under control of the *Thy1* promoter	Aβ40 and Aβ42 accumulation and amyloid plaques (onset: 2 months of age; more severe in females), gliosis, neuron loss (onset: 6 months of age), synaptic degeneration (onset: 9 months of age)	Impaired general memory function and spatial working memory (onset: 4 months of age), abnormal olfactory-guided behaviors (onset: 6 months of age), motor impairments (onset: 9 months of age).	[22, 25]
THY-Tau22	Overexpression of human *MAPT* gene (with G272V and P301S variants) under control of the *Thy1.2* promoter	Hyperphosphorylation of the tau protein, neurofibrillary-like tau inclusions, and ghost tangles (onset: 3 months of age)	Learning and memory diffculties, including deficits in spatial, social, and contextual learning (onset: 6 months of age)	[23]
3xTg-AD	Overexpression of human *APP* (with K670N and M671L variants) and *MAPT* (with P301L variant) genes, and the M146V variant in the endogenous *Psen1* gene	Amyloid plaques (onset: 3 months of age), neurofibrillary tangles (onset: 12 months of age)	Cognitive impairment with deficits in spatial and contextual paradigms (onset: 3 months of age), learning and memory deficits (onset: 6 months of age)	[24]

The 5xFAD model reflects many of the memory, cognitive, and neurochemical dysfunctions observed in familial AD [22]. The model is characterized by the presence of mutations in the *APP* (K670N, M671L, I716V, V717I) and *PSEN1* (M146L and L286V) genes and by overproduction of Aβ, due to expression of both transgenes from the *Thy1* promoter. The genetic background of this line is C57BL/6J. Aβ deposits appear around 14 weeks of age. Aβ42 and APP levels are significantly elevated relative to controls starting at 4 months of age. These changes become more severe at 12 months of age and involve both the cortex and hippocampus. Moreover, significantly increased levels of the soluble form of Aβ40 and Aβ42 are observed in plasma and brain lysates [25].

In the THY-Tau22 model, there are G272V and P301S mutations in the *MAPT* gene of mice with the C57BL/6J genetic background [23]. The mice exhibit numerous tauopathies, including tau protein hyperphosphorylation and the formation of neurofibrillary inclusions and tangles. The p-tau levels are slightly lower in the hippocampus, and only trace levels are detected in the spinal cord. The first symptoms are visible at 3 months of age, with a significant increase 6 months later. These mice have learning and memory difficulties, including deficits in spatial, social, and contextual learning that cannot be attributed to motor impairments [23].

The 3xTg-AD model contains mutations in the *APP* gene (K670N, M671L), as well as in genes coding for presenilin 1 (M146V) and tau protein (P301L) [24]. Amyloid plaques form from the age of 3 months, first in the cortex and then in the hippocampus. Neurofibrillary tangles appear around 12 months of age, initially in the hippocampus and then in the cortex. This model also demonstrates age-related synaptic dysfunctions, as well as progressive impairments in spatial memory, beginning at 4 months of age, and deficits in passive avoidance [24].

In the case of 5xFAD, the line was maintained by backcrossing to B6SJLF1 at every generation. Therefore, C57BL/6J and SJL/J were also segregated in the progeny. Specifically, a mixture of both 5xFAD males x WT SJL females and 5xFAD females x WT SJL males crosses was performed; thus, the experimental animals carried mixed transgene inheritance. This is also important when investigating the THY-Tau22 line (and 3xTg-AD mice too), since both lines also utilize the *Thy1* promoter to drive transgene expression. In the case of 3xTg-AD, B6129SF2/J mice might be potentially used as controls (these F2 (second filial generation) hybrid mice are the offspring of an F1 x F1 mating, itself the product of a cross between C57BL/6J females (B6) and 129S1/SvImJ males (129 S)). However, to maintain the same control line (potentially providing the most comparable results) across all AD genetic models, C57BL/6J was employed. Although this appeared to be a reasonable choice, it caused some limitations, as discussed in the ‘Discussion’ section. Because of this relatively complex experimental system, in all models used, mice from each generation were genotyped to distinguish genetic variants (homozygous mutations, heterozygous or wild-type allele(s) for relevant genes).

For animal housing, an animal room was used, ensuring ventilation with 15 air changes per hour. The size of each cage was 15 cm (high) x 400 cm^2^ (surface). Attractants and accessories for rodents were provided. The temperature was 22 ± 2 °C, humidity was maintained at 50 ± 5%, and the light/dark periods were 12/12 h. Animals had access to water and food *ad libitum*. Since 4′,5,7-trihydroxyisoflavone was the investigated molecule, fodder for mice with a reduced amount of isoflavones was used. The following legal requirements were fulfilled: (i) the recommendations of the European Commission on the welfare of animals used in scientific experiments, (ii) the Act on the Protection of Animals Used for Scientific or Educational Purposes (Journal of Laws, Poland, February 26, 2015). The experiments were approved by the Local Ethics Committee for the Care and Use of Laboratory Animals in Bydgoszcz, Poland (resolution no. 41/2022).

Each experimental group consisted of 6 mice (either 6 males or 6 females per group), according to calculations conducted with the Power analysis, using the following parameters: 1-β = 0.8 (statistical power), α = 0.05 (significance level), d = 0.7 (effect size). Animals (both sexes) belonging to control groups (C57BL/6J) and AD groups (5xFAD, THY-Tau22, and 3xTg-AD) were either untreated (no marks in figures) or treated daily (by oral administration through a metal gavage, 25 mm-long) with water (marked as W in figures) or 150 mg/kg/day genistein (marked as G in figures), respectively. Total volume of each administered liquid was 0.1 ml. Animals of the determined genotypes were randomly assigned to the indicated experimental groups. To blind the experiments, behavioral tests and biochemical analyses were conducted by researchers who did not know which groups included mutant or control animals or which treatment was applied. After all measurements were completed, the unblinding procedure was performed. No cases of animal death before euthanasia were noted in all experiments. Altogether, considering both genistein-treated and control mice, 216 animals were used in this study.

### The timeframe of interventions

**Fig. 1:**
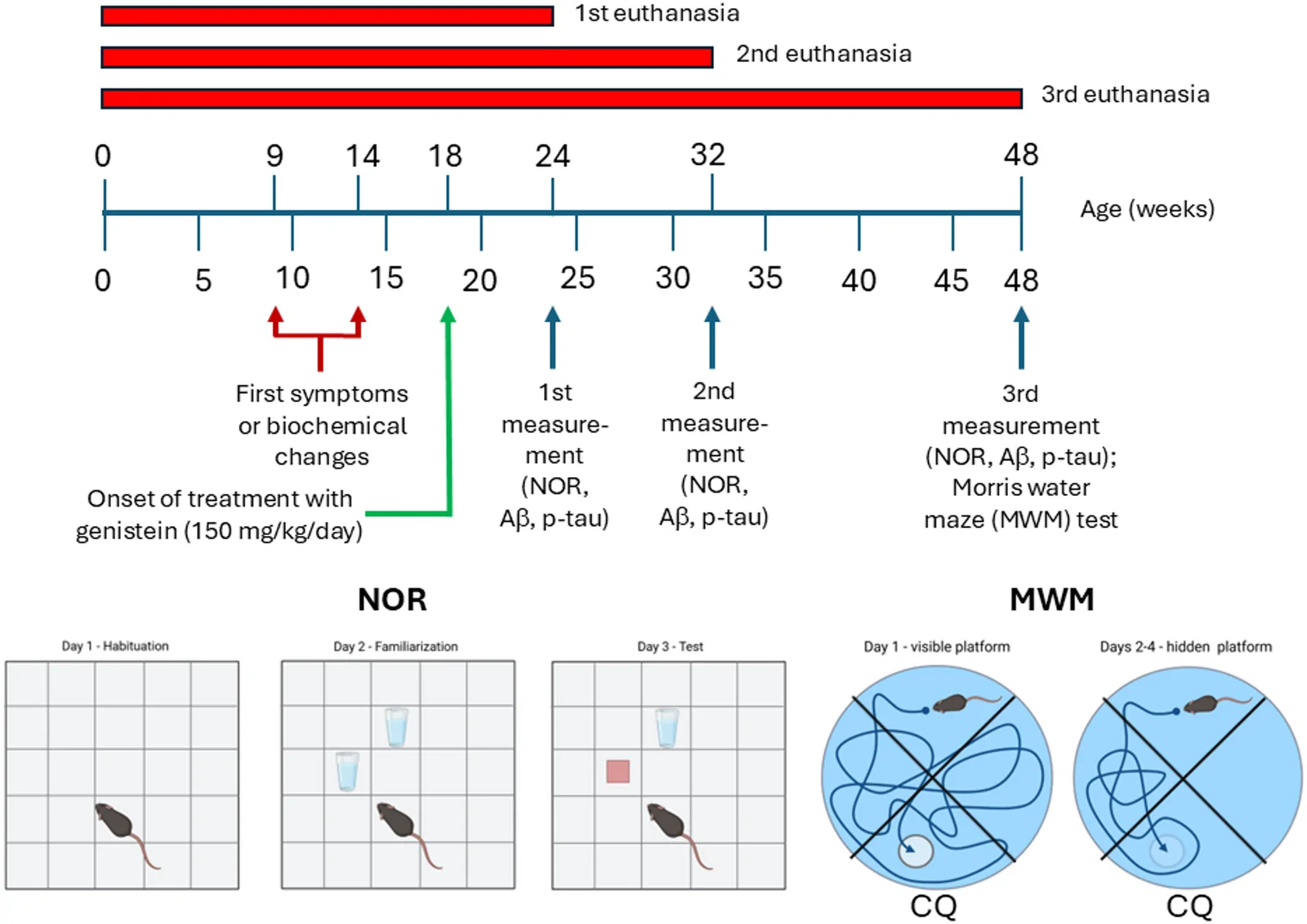
The scheme of the experiments. Murine models of AD (5xFAD, THY-Tau22, and 3xTg-AD) were used along with the control (C57BL/6J) mice. The timescale and major time points of experiments are shown. The first physiological symptoms or biochemical changes in these AD models appear between 9 and 14 weeks of age. Treatment with genistein (150 mg/kg/day) or water was initiated at the age of 18 weeks. Three measurements of levels of Aβ and p-tau (in plasma or brain homogenate) and novel object recognition (NOR) tests were conducted three times, when mice were 24, 32, and 48 weeks old; the Morris water maze (MWM) test was performed at the age of 48 weeks. Schemes of NOR and MWM tests are shown in the lower part of the figure. For obtaining brains to measure Aβ and p-tau levels, mice were subjected to euthanasia at the 1st, 2nd, and 3rd measurement timepoints. Each group of experimental animals consisted of 6 mice (male and female groups were separate experimental groups)

The timeframe of interventions is presented schematically in Fig. 1, while details of experimental procedures are described in subsequent subsections. Briefly, genistein (or the vehicle (water) in control experiments) was administered to mice orally (150 mg/kg daily), starting from 18 weeks of age, which was shortly after the appearance of either the first physiological symptoms or biochemical changes (9–14 weeks of age), depending on the model. Novel object recognition (NOR) tests were conducted at weeks 24, 32, and 48, while the Morris water maze test was performed at week 48. Samples for biochemical analyses (levels of Aβ and p-tau) were withdrawn from mice at 24, 32, and 48 weeks old. Since biochemical changes were also measured in brains, euthanasia was performed in part of the animals at the timepoints indicated above and shown in the upper part of Fig. 1.

### Morris water maze test

The protocol used in this study was adapted from previously published ones [13, 14]. A circular pool measuring 150 cm (diameter) x 60 cm (depth), with black walls and filled with water, was used. The pool was divided artificially into quadrants (using a recording camera coupled to a processing device (EthoVision XT; Noldus, The Netherlands)). A circular, white landmark of 10 cm in diameter was mounted on the wall of the pool, above a transparent platform (located just above or just under the water surface; see subsequent description for details). The quadrant indicated by this landmark was marked as CQ (Fig. 1). The experiments were conducted for four consecutive days. Every animal participating in the experiment was placed in the pool so that its head faced the pool wall in the quadrant opposite that with the landmark. On day 1, the training session was performed, during which the platform was placed 1 cm above the water surface. On days 2, 3, and 4, the same platform was placed 1 cm below the water surface. The movement of each mouse was recorded by the camera, and the distance travelled to the platform and the time needed to reach it (latency to platform) were measured.

### Novel object recognition test

The protocol used in this study was adapted from a previously published one [14]. A square arena measuring 100 × 100 cm was used. The floor was divided into 25 squares (equal in size). The arena was surrounded by walls of the height of 60 cm. For experiments, 3 objects were used and placed in the arena; two of them were identical, and one was different (for example, two identical cups and one cube that differed in color, shape, and texture). A mouse was put in one corner of the arena, inside a square called the starting square. At the opposite side of the arena (the opposite square), the objects were placed (Fig. 1). A video camera coupled to an automatic tracking system (EthoVision XT; Noldus, Netherlands) was used for recording animal movements. After each recording, the arena was cleaned with 70% ethanol to eliminate any potential odor effects on animal behavior. The entire experiment duration was 3 days. On the first day, mice were placed in an arena devoid of any objects for 10 min to familiarize them with the environment. On the second day, mice were placed in the arena analogously (again for 10 min of exploration), but the two identical objects were added. On the third day, animals were placed into the arena containing both familiar and novel (different) objects, and exploration was conducted again for 10 min. Based on recording times spent near particular objects, time spent near the novel object was calculated.

### Blood collection

Samples of blood were obtained under anesthetic conditions, using 87.5 mg/kg ketamine and 12.5 mg/kg xylazine, applied in a volume of 0.1 ml per mouse. A capillary sputtered with EDTA was employed, and blood was collected from the venous plexus inside the orbit behind the eyeball. Plasma was obtained after centrifugation (400 x *g*, 10 min, 4 °C), and samples were stored at -80 °C.

### Euthanasia and brain tissue isolation

Before euthanasia, animals were anesthetized with 2.5% isoflurane (applied through inhalation with a flow rate of 0.5 l/min). Then, pentobarbital was administered intraperitoneally at a dose of 120 mg/kg. Brain tissue was isolated and subjected to homogenization with Bullet BlenderTM (Next Advance, #4116-BBY24M-CE), using 0.2 ml of the T-PER Tissue Protein Extraction Reagent (ThermoFisher Scientific, #78510).

### Determination of levels of Aβ40, Aβ42, and p-tau

Levels of Aβ40, Aβ42, and p-tau (phosphorylated at the S199 residue) proteins in plasma and brain homogenates were determined using ELISA assays with specific antibodies (ThermoFisher Scientific, #KMB3481, #KMB3441, and #KMB7041, respectively). The quantification of results obtained in sandwich ELISA assays was based on measurements of the signals (in a microplate reader) formed as effects of reactions with specific substrates, catalyzed by enzymes coupled to secondary antibodies, according to the manufacturer’s instructions. Intensities of the signals were directly proportional to the concentrations of targets present in the samples.

### Statistical analysis

The results are presented as mean values ± standard deviation (SD). For statistical analyses, the SPSS 21.0 software (SPSS Inc., Armonk, USA) was used. Normality of the distribution of variables and homogeneity of the variances were investigated using the Kolmogorov-Smirnov and Levene tests, respectively. Two-way ANOVA and Tukey’s post hoc tests were employed. Differences were considered significant when *p* < 0.05.

## Results

### The murine models of AD and experimental design

To assess the efficacy of 4′,5,7-trihydroxyisoflavone in well-defined animal models of AD, we have employed three murine models, 5xFAD [22], THY-Tau22 [23], and 3xTg-AD [24], along with the control mouse line (C57BL/6J). They are described in more detail in the Methods section, but their crucial characteristics are that 5xFAD overproduces Aβ, THY-Tau22 contains increased levels of p-tau, while 3xTg-AD reveals increased levels of both Aβ and p-tau. In all these murine models of AD, the first physiological symptoms or biochemical changes appear at 9–14 weeks of age (Table 1). The scheme of experiments described in this report is presented in Fig. 1. Despite differences in the course of the disease between the used murine models (see Table 1 for details), the same timepoints were used in all experiments to keep the maximal possible coherence of the whole study. The onset of administration of either genistein (at a dose of 150 mg/kg/day, as in previous studies [13, 14]) or water (control) began at 18 weeks of age. Since no significant differences could be observed in any measured parameters between untreated or water-treated mice (indicating no adverse effects of the administration procedure), the term “untreated” will be used further in the text to indicate both those experimental conditions. When spatial memory was assessed using the Morris water maze test, the experiments were performed at the age of 48 weeks. To assess learning capabilities and long-term memory, the novel object recognition assay was conducted three times, when animals were 24, 32, and 48 weeks old. At the same timepoints, levels of Aβ40, Aβ42, and p-tau were measured in plasma or brain homogenates of mice belonging to relevant models. Such a schedule of experiments (presented schematically in Fig. 1) allowed us to estimate the effects of genistein on both cognitive functions (spatial memory, learning, and long-term memory) and biochemical parameters (levels of Aβ40, Aβ42, and p-tau) in murine genetic models of AD.

### Efficacy of genistein in improving spatial memory

**Fig. 2:**
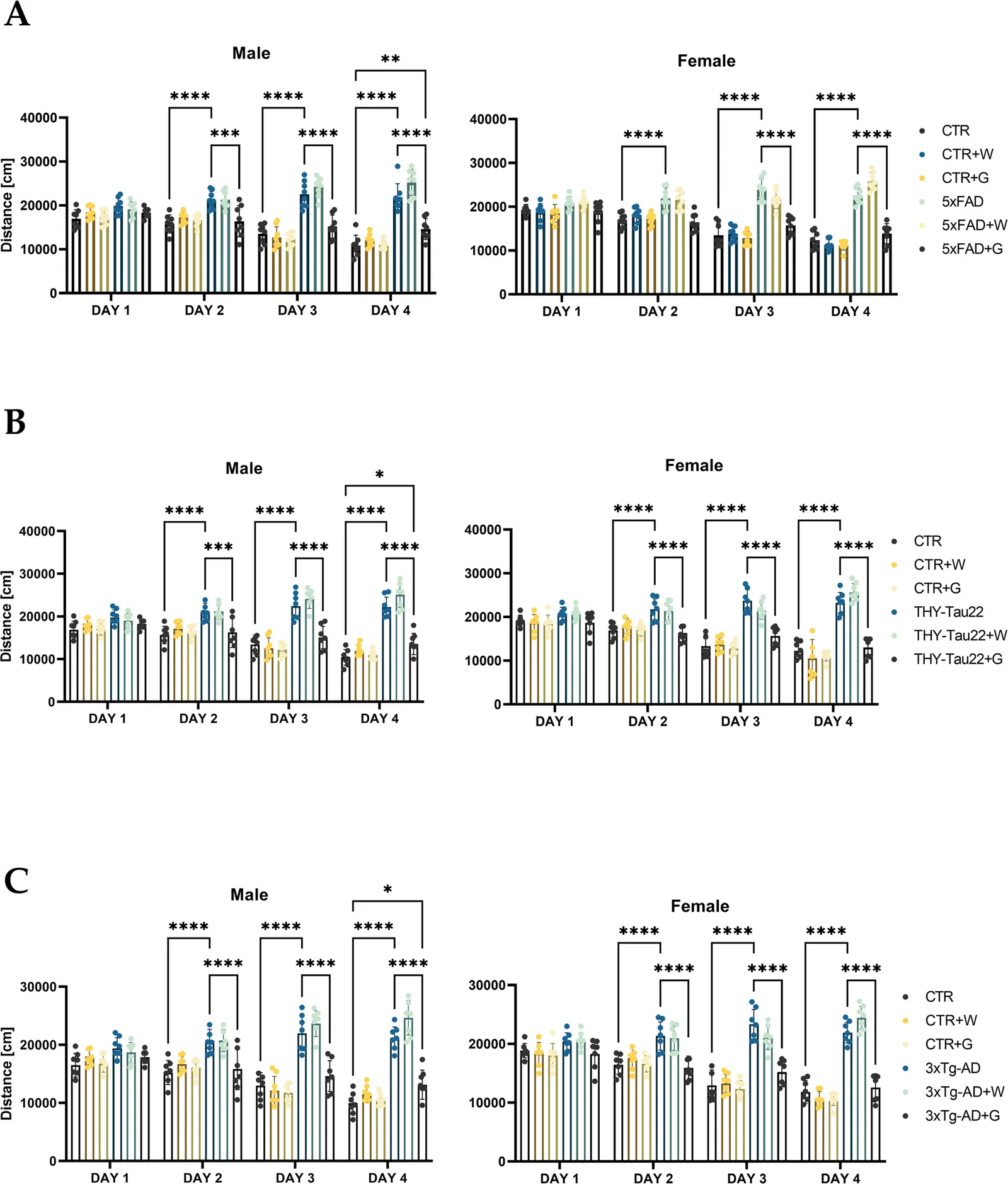
Effect of genistein on spatial memory as assessed by measuring the distance travelled to the platform in the Morris water maze (MWM) test. Murine models of AD, 5xFAD (**A**), THY-Tau22 (**B**), and 3xTg-AD (**C**), were used along with the control (CTR; C57BL/6J) mice. Males (*n* = 6; left panels) and females (*n* = 6; right panels) were employed as separate groups. Animals were either untreated (no sign) or treated with water (W) or genistein (G; at 150 mg/kg/day) starting at the age of 18 weeks. The distance travelled to reach the platform in the pool was measured (in cm) over the consecutive four days of the experiment, performed with mice at the age of 48 weeks. The results are presented as mean values ± SD, with demonstration of each individual data point. Statistically significant differences (in two-way ANOVA with Tukey’s post hoc test) between particular groups of animals are marked as follows: *, *p* < 0.05; **, *p* < 0.01; ***, *p* < 0.001; ****, *p* < 0.0001. Details of statistical analyses are presented in Supplementary Table S1

To assess the spatial memory of animals, the Morris water maze test we used. The test was performed once, when mice were 48 weeks old, to avoid any potential influence of long-term memory effects (a potential memory retention of the platform position); long-term memory was estimated using another assay (the novel object recognition test, which allows changing of the recognized objects). The measured parameters were the distance travelled by animals to the platform and the latency to the platform, both indicating spatial memory effectiveness. Despite the difference in the course of the disease, in all three animal AD models used, untreated animals revealed significantly impaired spatial memory relative to control (wild-type) mice, as assessed by measuring the distance they required to find the platform, especially at later days of the experiment, when healthy animals remembered the location of the platform better and better (Fig. 2). However, oral administration of genistein (since week 18) to 5xFAD mice caused significant improvement in spatial memory; the same conclusions can be drawn for THY-Tau22, and 3xTg-AD models (Fig. 2). Interestingly, results obtained for females of all three AD models were statistically indistinguishable from those observed in control animals (indicating correction of this cognitive parameter); genistein-treated AD males improved considerably relative to untreated AD animals, while still revealing some deficiency when compared to wild-type controls (Fig. 2).

**Fig. 3:**
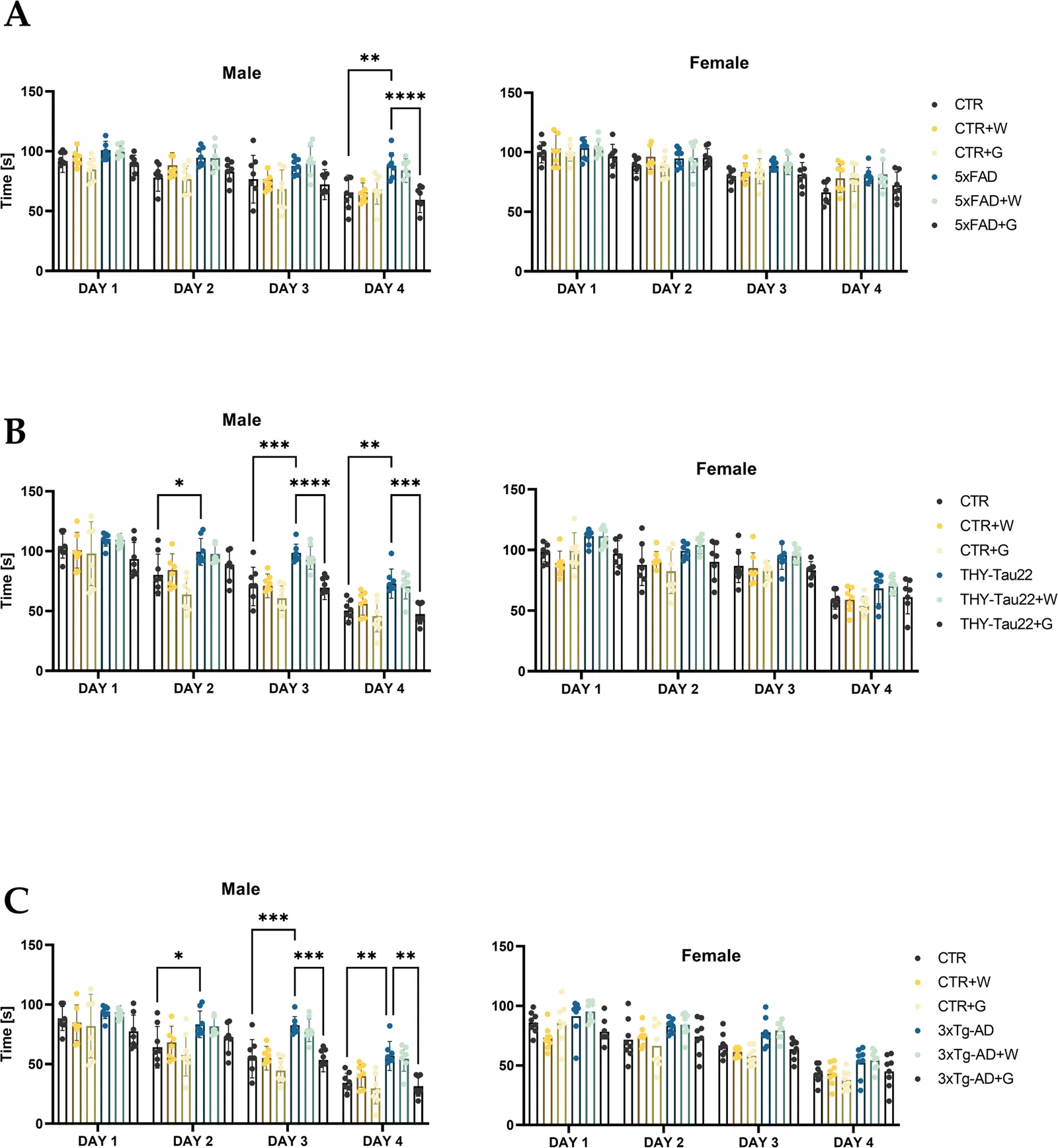
Effect of genistein on spatial memory as assessed by measuring the latency to the platform in the Morris water maze (MWM) test. Murine models of AD, 5xFAD (**A**), THY-Tau22 (**B**), and 3xTg-AD (**C**), were used along with the control (CTR; C57BL/6J) mice. Males (*n* = 6; left panels) and females (*n* = 6; right panels) were employed as separate groups. Animals were either untreated (no sign) or treated with water (W) or genistein (G; at 150 mg/kg/day) starting at the age of 18 weeks. Time spent to reach (i.e., latency to) the platform in the pool was measured (in seconds) over the consecutive four days of the experiment, performed with mice at the age of 48 weeks. The results are presented as mean values ± SD, with demonstration of each individual data point. Statistically significant differences (in two-way ANOVA with Tukey’s post hoc test) between particular groups of animals are marked as follows: *, *p* < 0.05; **, *p* < 0.01; ***, *p* < 0.001; ****, *p* < 0.0001. Details of statistical analyses are presented in Supplementary Table S1

The efficacy of 4′,5,7-trihydroxyisoflavone in improving spatial memory deficits in 5xFAD, THY-Tau22, and 3xTg-AD models was confirmed by measuring latency to the platform. Although less spectacular than the measurement of distance travelled to the platform, shortening of the time needed to find the platform was also evident in genistein-treated AD male mice (Fig. 3). Nevertheless, in the case of 5xFAD males, significant effects were observed only on day 4 of the experiment, contrary to those of THY-Tau22 and 3xTg-AD, where the genistein-dependent improvement was also noted earlier (Fig. 3). In contrast, no statistically significant differences were observed between the female groups of all three AD models (Fig. 3). Therefore, in this specific parameter, effects of genistein treatment were more pronounced in AD males than in females. However, one should note that females of 5xFAD, THY-Tau22 and 3xTg-AD did not differ from wild-type females in this particular measurement; thus, one should not conclude about a lack of efficacy of genistein in improving latency to the platform.

In summary, the Morris water maze test indicated that administration of 4′,5,7-trihydroxyisoflavone could improve the spatial memory deficit in 5xFAD, THY-Tau22, and 3xTg-AD genetic models of AD, otherwise evident in untreated animals. Despite some differences between particular models and between males and females, the positive effects of genistein on spatial memory in AD animals were evident.

### Efficacy of genistein in improving learning capability and long-term memory

**Fig. 4:**
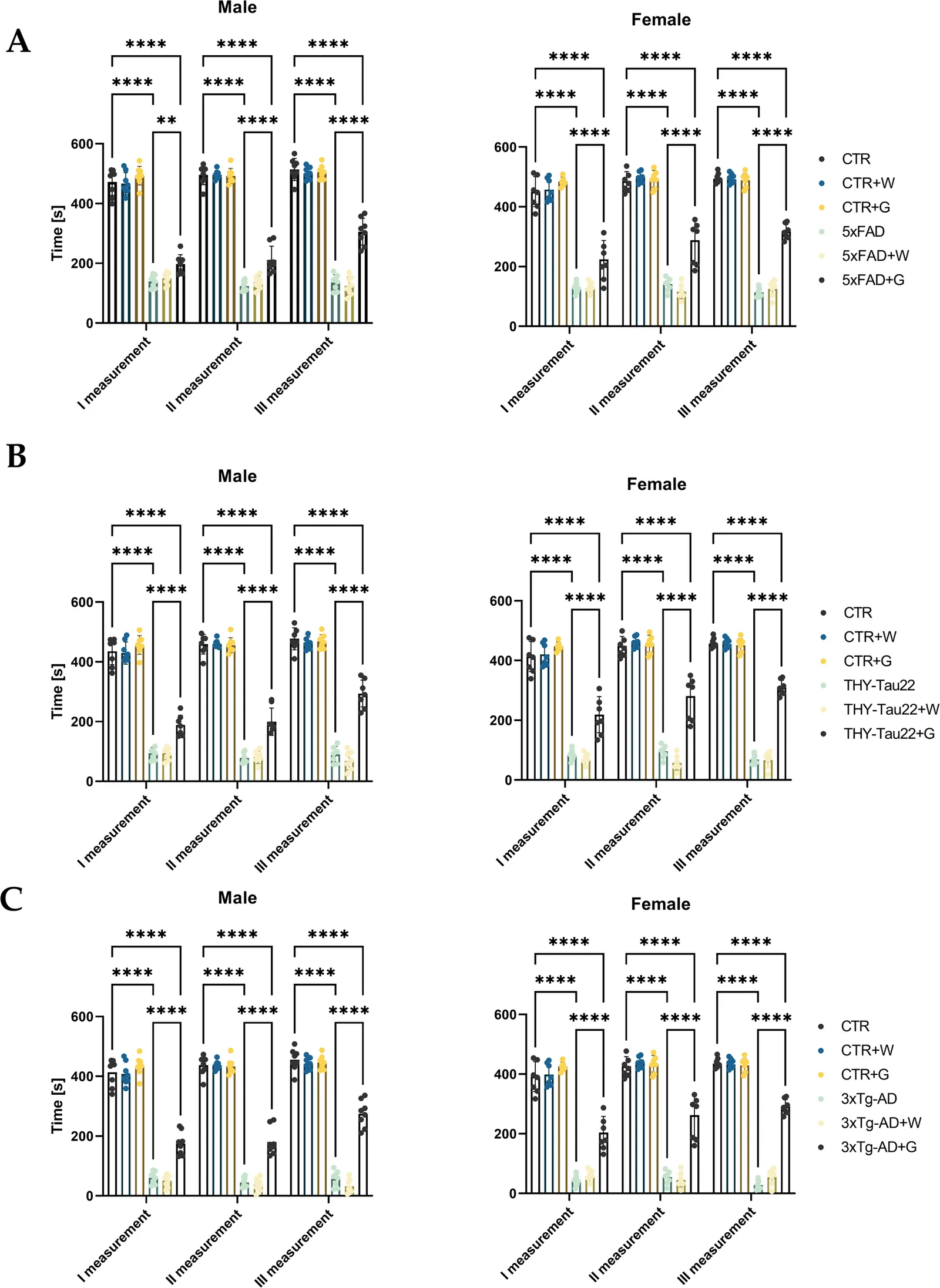
Effect of genistein on learning and long-term memory as assessed by measuring the time spent near the novel object in the novel object recognition (NOR) test. Murine models of AD, 5xFAD (**A**), THY-Tau22 (**B**), and 3xTg-AD (**C**), were used along with the control (CTR; C57BL/6J) mice. Males (*n* = 6; left panels) and females (*n* = 6; right panels) were employed as separate groups. Animals were either untreated (no sign) or treated with water (W) or genistein (G; at 150 mg/kg/day) starting at the age of 18 weeks. Time spent near the novel object (in seconds) during the 10-min session was measured during the third day of the experiment (after familiarization of animals to objects treated as “known”). The experiments were conducted three times during the study, when mice were at the age of 24 (I measurement), 32 (II measurement), and 48 (III measurement) weeks. The results are presented as mean values ± SD, with demonstration of each individual data point. Statistically significant differences (in two-way ANOVA with Tukey’s post hoc test) between particular groups of animals are marked as follows: **, *p* < 0.01; ****, *p* < 0.0001. Details of statistical analyses are presented in Supplementary Table S1

To test the effects of 4′,5,7-trihydroxyisoflavone on the abilities of AD animals to learn new things and to assess their long-term memory, the novel object recognition assay was employed. In this assay, during the 3-day session (10 min exploration each day), mice were exposed to the presence of known and novel objects. The time spent near the novel object on day 3 is an indicator of both the learning capabilities as well as long-term memory (as the animals should remember, from the previous day experience, which object is already known and which is new). The experiments were performed three times, at the ages of 24, 32 and 48 weeks (measurements I, II and III, respectively), to assess the time-course of genistein effects. In this test, the potential memory retention effects from previous experiments could be excluded by using different known and novel objects in each set of experiments. The results presented in Fig. 4 indicate that administration of genistein significantly improved learning capability and long-term memory in 5xFAD, THY-Tau22 and 3xTg-AD animals (all three genetic AD models revealed the same tendency). Although the results of AD mice did not reach those measured in control (wild-type) mice, the improvement relative to untreated AD animals was evident. The overall efficacy of the tested compound was similar in males and females of 5xFAD, THY-Tau22 and 3xTg-AD.

To sum up, administration of 4′,5,7-trihydroxyisoflavone could significantly improve, though not normalize, learning and long-term memory deficits in murine genetic models (5xFAD, THY-Tau22, and 3xTg-AD) of AD.

### Efficacy of genistein in reducing Aβ40, Aβ42, and p-tau levels

Since overproduction of Aβ and/or p-tau is the main cause of the appearance of symptoms in the animal models of AD employed in this study, levels of the curial pathogenic forms of Aβ (Aβ40 and Aβ42) and p-tau were determined in 5xFAD, THY-Tau22, and 3xTg-AD mice and compared to those occurring in control (wild-type) animals. The effects of genistein (administered from 18 weeks of age) were assessed when the animals were 24, 32, and 48 weeks old (measurements I, II, and III, respectively).

**Fig. 5:**
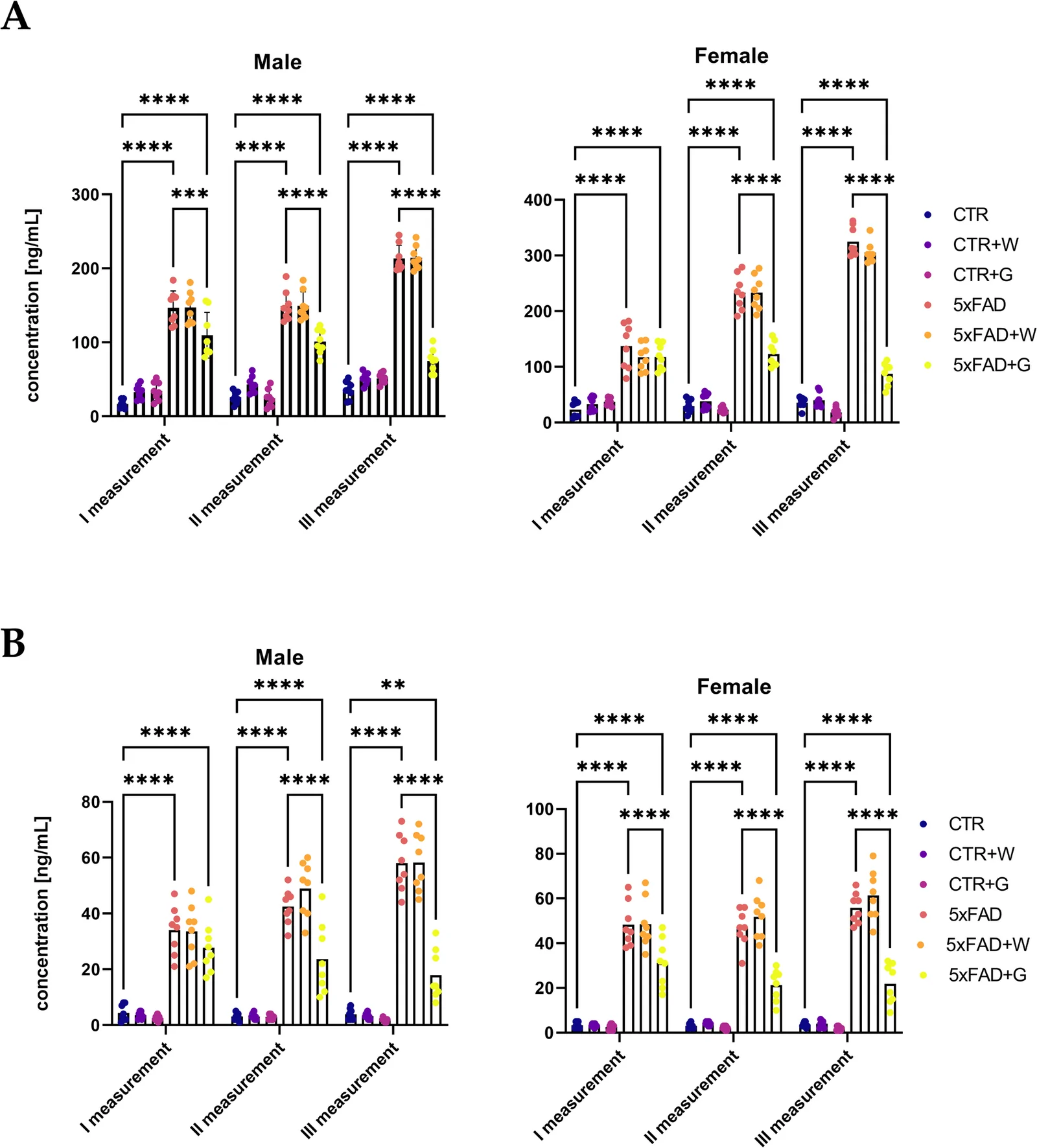
Treatment with genistein reduces levels of Aβ40 and Aβ42 in plasma of the 5xFAD animal AD model. AD mice (5xFAD) were used along with the control (CTR; C57BL/6J) mice. Males (*n* = 6; left panels) and females (*n* = 6; right panels) were employed as separate groups. Animals were either untreated (no sign) or treated with water (W) or genistein (G; at 150 mg/kg/day) starting at the age of 18 weeks. Levels of Aβ40 (**A**) and Aβ42 (**B**) were measured in plasma of mice. The experiments were conducted three times during the study, when mice were at the age of 24 (I measurement), 32 (II measurement), and 48 (III measurement) weeks. The results are presented as mean values ± SD, with demonstration of each individual data point. Statistically significant differences (in two-way ANOVA with Tukey’s post hoc test) between particular groups of animals are marked as follows: **, *p* < 0.01; ***, *p* < 0.001; ****, *p* < 0.0001. Details of statistical analyses are presented in Supplementary Table S1

**Fig. 6:**
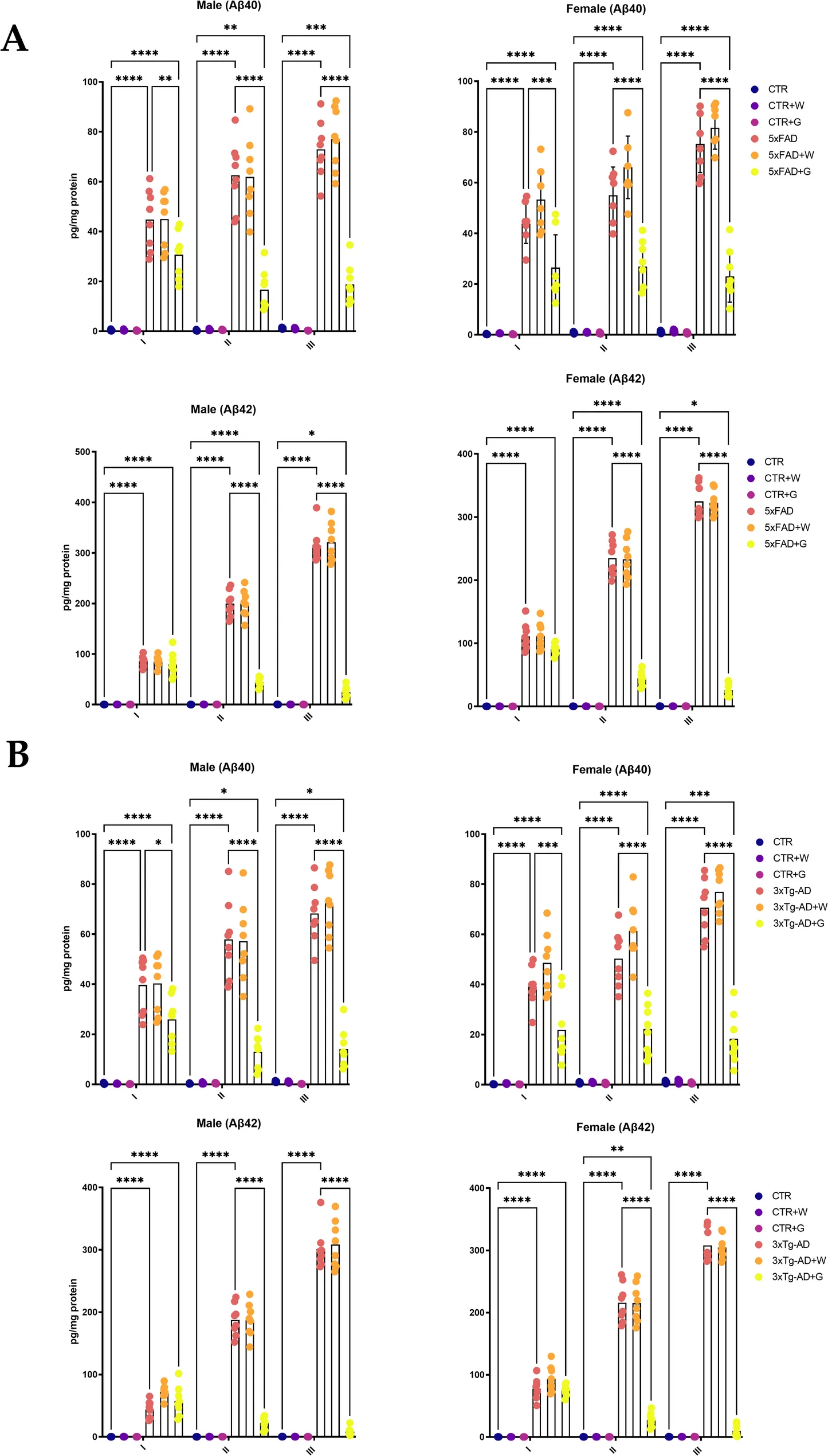
Treatment with genistein reduces levels of Aβ40 and Aβ42 in brain homogenates of animal AD models. Murine models of AD, 5xFAD (**A**) and 3xTg-AD (**B**), were used along with the control (CTR; C57BL/6J) mice. Males (*n* = 6; left panels) and females (*n* = 6; right panels) were employed as separate groups. Animals were either untreated (no sign) or treated with water (W) or genistein (G; at 150 mg/kg/day) starting at the age of 18 weeks. Levels of Aβ40 and Aβ42 were measured in plasma or brain homogenates of mice. The experiments were conducted three times during the study, when mice were at the age of 24 (I measurement; marked as I), 32 (II measurement; marked as II), and 48 (III measurement; marked as III) weeks. The results are presented as mean values ± SD, with demonstration of each individual data point. Statistically significant differences (in two-way ANOVA with Tukey’s post hoc test) between particular groups of animals are marked as follows: *, *p* < 0.05; **, *p* < 0.01; ***, *p* < 0.001; ****, *p* < 0.0001. Details of statistical analyses are presented in Supplementary Table S1

Levels of Aβ40 were measured in 5xFAD and 3xTg-AD mice (the THY-Tau22 model was excluded as it overproduces p-tau, but not Aβ), and compared to controls. As expected, a systematic increase in Aβ40 levels in plasma (in 5xFAD mice) and brain homogenates (in both tested models) was observed in untreated animals (Figs. 5 and 6, respectively). However, administration of genistein (starting at 18 weeks of age) significantly reduced the accumulation of these forms of Aβ, in a time-related manner, with higher efficacy at later times (Figs. 5 and 6). No significant differences were observed between males and females of the 5xFAD and 3xTg-AD lines (Figs. 5 and 6). Similar results were obtained when levels of Aβ42 were measured in brain homogenates from 5xFAD and 3xTg-AD, though the effect of the duration of the treatment with genistein was even more pronounced (Fig. 6).

**Fig. 7:**
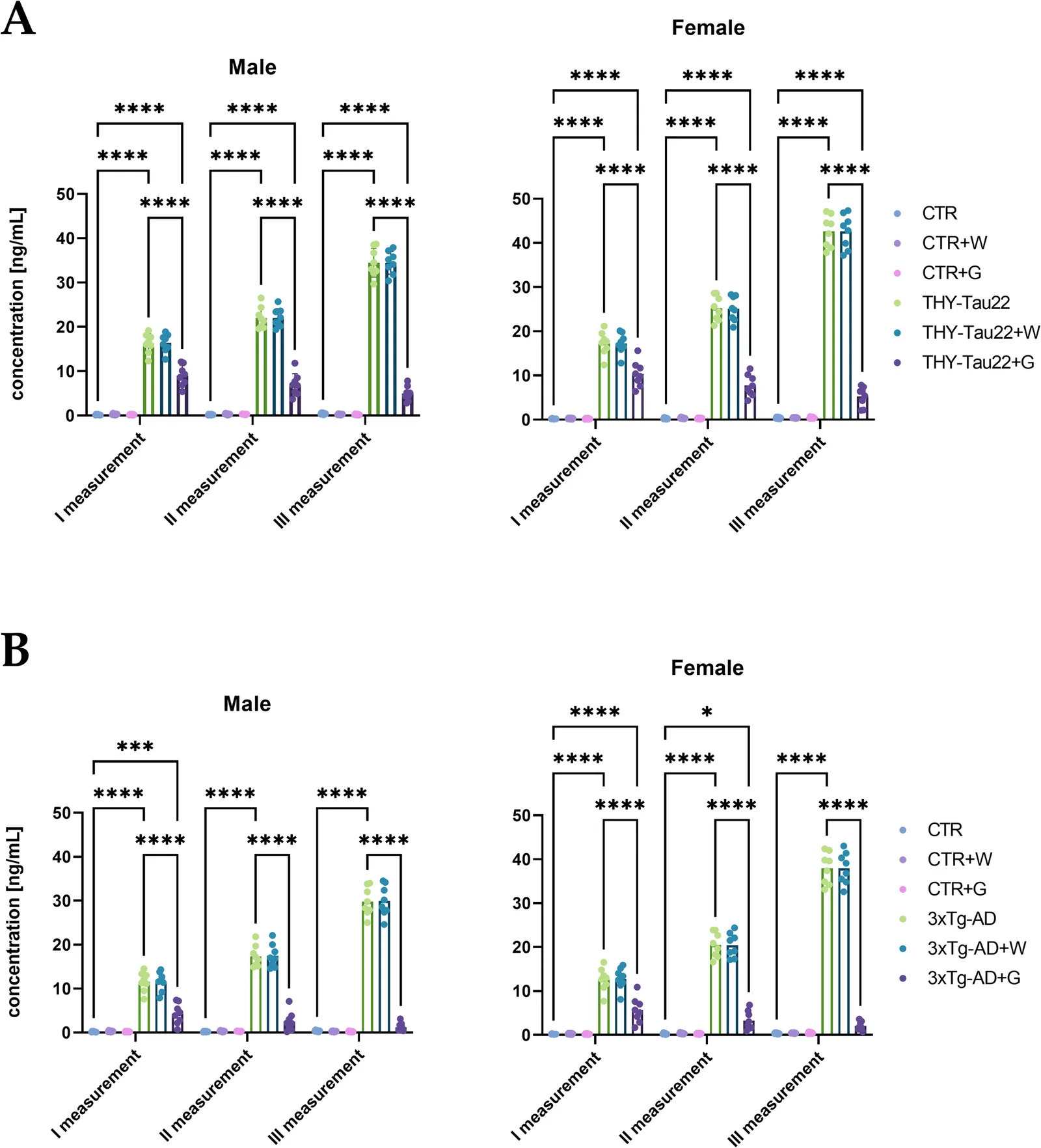
Treatment with genistein reduces levels of hyperphosphorylated tau protein (p-tau) in brain homogenates of animal AD models. Murine models of AD, THY-Tau22 (**A**) and 3xTg-AD (**B**), were used along with the control (CTR; C57BL/6J) mice. Males (*n* = 6; left panels) and females (*n* = 6; right panels) were employed as separate groups. Animals were either untreated (no sign) or treated with water (W) or genistein (G; at 150 mg/kg/day) starting at the age of 18 weeks. Levels of hyperphosphorylated tau protein (p-tau) were measured in brain homogenates. The experiments were conducted three times during the study, when mice were at the age of 24 (I measurement), 32 (II measurement), and 48 (III measurement) weeks. The results are presented as mean values ± SD. Statistically significant differences (in two-way ANOVA with Tukey’s post hoc test) between particular groups of animals are marked as follows: *, *p* < 0.05; **, *p* < 0.01; ***, *p* < 0.001; ****, *p* < 0.0001. Details of statistical analyses are presented in Supplementary Table S1

Effects of genistein on levels of p-tau were investigated in THY-Tau22 and 3xTg-AD mice (5xFAD mice do not accumulate p-tau) and only in brain homogenates (p-tau does not accumulate in blood). In both tested models, administration of 4′,5,7-trihydroxyisoflavone drastically reduced levels of p-tau in brains, and the effects were more pronounced with a longer treatment (Fig. 7). No significant differences between sexes were noted.

The results presented in this subsection provided evidence that administration of 4′,5,7-trihydroxyisoflavone significantly reduced levels of Aβ and/or p-tau in murine genetic models of AD. This applied to all tested animal models, 5xFAD, THY-Tau22, and 3xTg-AD. Although the results of the measurements of Aβ and p-tau levels in brain homogenates might directly correlate with neurological dysfunctions and their corrections, the concentration of Aβ forms in plasma could also be valuable as a marker indicating efficacy of the tested compound in lowering the abundance of these pathological forms of amyloid molecules.

## Discussion

Recent approval of two anti-AD therapies, based on the use of anti-Aβ antibodies, provided a great hope for patients suffering from AD and their families. However, these expectations were significantly diminished in the light of the very recent, large-scale analysis of the efficacy of Aβ-targeting monoclonal antibodies [26]. In that work, based on efficacy estimates reported for over 20,000 patients, it was concluded that such antibodies revealed “little to no difference” relative to placebo when assessing cognitive functions, dementia severity, and functional ability. The authors of that report concluded that despite “successful removal of Aβ from the brain”, the therapeutic effect of anti-AD antibodies is “trivial” [26]. Therefore, eliminating Aβ accumulation appears to be insufficient to obtain clinically significant effects. In this light, searching for alternative anti-AD therapies seems to be of high importance.

The use of genistein, a naturally occurring isoflavone (specifically, 4′,5,7-trihydroxyisoflavone), has been proposed as one of the possible alternative strategies for AD treatment [27]. Since this compound was found to be an efficient autophagy stimulator [10, 14], while at the same time revealing a beneficial safety profile [28], it was assumed that genistein might be effective in eliminating toxic aggregates that accumulate in AD. While Aβ and p-tau are major components of pathological aggregates (and may facilitate their formation), the “mature” insoluble complexes also contain other macromolecules, like lipids, sugars, and nucleic acids [29–32]. However, since autophagy depends on the action of lysosomes containing hydrolases that can degrade all kinds of biological macromolecules, this process is effective in decaying not only single molecules but also aggregates or even entire damaged organelles [33].

The assumptions presented above led us to test the effects of genistein on AD using an animal model of the sporadic form of this disease [13]. The streptozotocin-induced “brain diabetes” resulted in accumulation of Aβ and p-tau in the brains of rats, resembling the course of human AD. Using such a model, it was indicated that behavioural parameters (including memory, anxiety, and others) were significantly improved in AD rats treated with genistein relative to untreated AD animals [13].

Although those results were promising, the streptozotocin-induced AD model has some important limitations, such as low specificity (causing various effects, not directly linked to AD, like diabetes), an invasive method of toxin administration, and a time-course significantly different from that observed in naturally occurring AD. Therefore, to comprehensively assess the effects of the tested compound as a potential anti-AD drug, we decided to employ well-defined genetic murine models of this disease. In this report, using 5xFAD, THY-Tau22, and 3xTg-AD models, we were able to demonstrate that orally administered genistein could improve spatial memory, learning capabilities, and long-term memory in the affected animals, as well as cause a reduction in the levels of Aβ40, Aβ42, and p-tau in plasma and/or brains of these mice. Despite differences between these animal genetic models of AD, including different direct causes and time-courses of the diseases, as well as differences between males and females, in virtually all tests and measurements conducted in this study, genistein revealed efficacy in ameliorating the AD-related symptoms. These are promising results, encouraging further work on testing 4′,5,7-trihydroxyisoflavone as an anti-AD compound, and if these properties are confirmed, also subsequent actions toward developing genistein-based anti-AD drug(s).

An intriguing point is the mechanism of genistein’s efficacy in alleviating AD-specific symptoms in the animal models, which is correlated with reduction in Aβ40, Aβ42, and p-tau levels. As indicated above, stimulation of autophagy might be the primary and/or major mechanism, leading to the elimination of toxic aggregates, composed of different macromolecules [13]. On the contrary, genistein has also been used previously in other studies involving various AD models, indicating that it might have beneficial effects. Interestingly, those reports considered different aspects of genistein activities, unrelated to autophagy stimulation. Instead, they focused on the effects of genistein on *ApoE*^*−/−*^ mice fed with a high-fat diet [34, 35], the modulation of apoptosis by this isoflavone [36], its anti-inflammatory activity [37] and its antioxidative properties [38]. Moreover, significant changes in the microbiome have been reported in both animals and humans with AD [19, 20], and these differences in gut microbiota can significantly affect neurological disorders via the gut-brain axis [39, 40]. Importantly, genistein was found to positively influence the gut microbiome by differentially modulating the growth of various bacterial species and the function of the gut barrier, which, if impaired, can also be a risk factor for AD [15–18]. It was also postulated that accumulation of heparan sulfate, one of the glycosaminoglycans, due to either its enhanced synthesis or impaired degradation, was s a crucial event in the early pathomechanism of AD [41]. Intriguingly, genistein can bind to the epidermal growth factor receptor [42], thus causing down-regulation of expression of genes coding for glycosaminoglycan synthetases [43], subsequently leading to partial inhibition of production of glycosaminoglycans (including heparan sulfate) [44]. Therefore, genistein-mediated impairment of heparan sulfate synthesis might be another mechanism by which this isoflavone could reveal beneficial effects, significantly amerliorate the course of AD.

Another aspect of anti-AD approaches is a dilemma whether any potential drug could reveal therapeutic effects when Aβ and p-tau deposits are already accumulated in high amounts, which can be observed in patients with already evident disease symptoms. Thus, one might ask whether such symptoms could be reversed or only some preventive effects might be expected when a drug is administered at very early stages of AD. In this light, one might speculate that anti-Aβ antibodies might reveal the highest efficacy when administered long term and possibly at lower dosages before even clinical symptoms appear. A question can also be asked whether genistein can be considered as an AD-drug or rather a disease-preventative agent, perhaps used for a long time, starting at a middle age. The latter proposal might be supported by results from a recently published study demonstrating the effects of genistein in preventing progressive loss of cognitive functions in mice treated with this compound for several months [45]. In contrast, in this report the onset of genistein treatment occurred after the appearance of the first physiological symptoms or biochemical changes, still demonstrating efficacy in partial reversion of these symptoms and in reducing levels of Aβ and p-tau. Therefore, the question about the possibility of effective treatment of AD at advanced AD stages or only efficient prevention of the disease, by either anti-Aβ antibodies, genistein, or any other drugs, remains open.

The main limitations of this study are the use of three different genetic models of AD, mixed transgene inheritance in the mice used in the experiments, and constraints of the behavioral and biochemical analyses. Namely, the genetic models reflect the mechanisms of familial AD rather than the sporadic form. To maintain the maximal consistency of this study, treatments were initiated at the same age for all animals. However, the three AD strains differ considerably in transgenicity, and therefore the way in which AD-like disease progresses differs for each mouse strain (see Table 1 for details). Moreover, the same control line, C57BL/6J, was used in experiments with all AD models to make the experimental results comparable to the greatest extent. However, this line is not an optimal control for all genetic AD models, especially 3xTg-AD. This is because C57BL/6J does not reproduce the 129 component present in the 3xTg-AD background. It is generally believed that for models based on the B6;129 S background, a more suitable approximate control strain would be B6129SF2/J. However, it should be noted that B6129SF2/J is also not a perfect genetic equivalent for 3xTg-AD, as the F2s have segregating alleles and are only an approximate match to the B6;129 background. Otherwise, one should note that effects of genistein were basically similar in all AD murine genetic models employed in this study; moreover, the results presented in this report are similar to those reported with the use of streptozotocin-induced model of sporadic AD [13], suggesting the general anti-AD effects (nevertheless, see a comprehensive discussion on benefits and limitations of the use of the streptozotocin-induced AD model [46]). However, one should take into consideration that behavioral tests conducted with animals cannot fully reflect human neurophysiology and cognitive parameters. Moreover, performed biochemical analyses, focused on the determination of levels of Aβ and p-tau, allowed the assessment of major, but not all biochemical changes occurring in human AD. Finally, histological analyses, including Aβ and p-tau pathology in particular brain regions, were not performed in this study. Furthermore, levels of autophagy-specific markers were not determined here. One might argue that despite previously reported demonstrations of genistein-mediated stimulation of autophagy, and the resultant reduction in the accumulation of various protein aggregates in different models [13, 14], such analyses would strengthen the conclusions presented in this work. Indeed, it is reasonable to propose that performing histological studies and conducting experiments aimed at demonstrating the efficiency of autophagy stimulation should be considered in further studies on the detailed molecular mechanisms of the effects of 4′,5,7-trihydroxyisoflavone on the course of AD.

In summary, this work demonstrates efficacy of 4′,5,7-trihydroxyisoflavone in improving cognitive parameters and lowering Aβ and p-tau levels in plasma and the brains of 5xFAD, THY-Tau22, and 3xTg-AD mouse lines, used as genetic models of AD. Nevertheless, the results presented in this report should be interpreted with caution, considering the above-described limitations.

## Conclusions

Genistein was shown to be effective in alleviating cognitive deficits and reducing levels of Aβ and p-tau in three murine genetic models of AD (5xFAD, THY-Tau22 and 3xTg-AD). This promising finding opens a way for further development of this isoflavone as a potential anti-AD drug. Moreover, this compound (4′,5,7-trihydroxyisoflavone) is a multifunctional agent and thus it might be considered as a molecule being compatible with the concept of using one drug for different purposes [47].

## Supplementary Information

Below is the link to the electronic supplementary material.

Supplementary Material 1

## Data Availability

The datasets with raw results obtained during this study are available from the corresponding author on reasonable request.

## Abbreviations

AD: Alzheimer's disease
Aβ: Amyloid beta
CNS: Central nervous system
p-tau: Hyperphosphorylated tau protein

## References

[1] Ju Y, Tam KY. Pathological mechanisms and therapeutic strategies for Alzheimer’s disease. Neural Regen Res. 2022;17:543–9. 10.4103/1673-5374.320970 PMC8504384 34380884

[2] Reitz C, Pericak-Vance MA, Foroud T, Mayeux R. A global view of the genetic basis of Alzheimer disease. Nat Rev Neurol. 2023;19:261–77. 10.1038/s41582-023-00789-z PMC10686263 37024647

[3] Hoogmartens J, Cacace R, Van Broeckhoven C. Insight into the genetic etiology of Alzheimer’s disease: A comprehensive review of the role of rare variants. Alzheimers Dement. 2021;13:e12155. 10.1002/dad2.12155 PMC7896636 33665345

[4] Feng JQ, Yang LL, Luo YX, Yao XQ. Alzheimer’s disease: from molecular pathways to therapies. Mol Biomed. 2026;7:43. 10.1186/s43556-026-00443-2 PMC13047008 41925951

[5] Hampel H, Cummings J, Blennow K, Gao P, Jack CR Jr, Vergallo A. Developing the ATX(N) classification for use across the Alzheimer disease continuum. Nat Rev Neurol. 2021;17:580–9. 10.1038/s41582-021-00520-w 34239130

[6] Oxford AE, Stewart ES, Rohn TT. Clinical trials in Alzheimer’s disease: A hurdle in the path of remedy. Int J Alzheimers Dis. 2020;2020:5380346. 10.1155/2020/5380346 PMC7152954 32308993

[7] Huang LK, Kuan YC, Lin HW, Hu CJ. Clinical trials of new drugs for Alzheimer disease: a 2020–2023 update. J Biomed Sci. 2023;30:83. 10.1186/s12929-023-00976-6 PMC10544555 37784171

[8] Gyimesi M, Okolicsanyi RK, Haupt LM. Beyond amyloid and tau: rethinking Alzheimer’s disease through less explored avenues. Open Biol. 2024;14:240035. 10.1098/rsob.240035 PMC11285855 38862019

[9] Tondo G, De Marchi F, Bonardi F, Menegon F, Verrini G, Aprile D, et al. Novel therapeutic strategies in Alzheimer’s disease: pitfalls and challenges of anti-amyloid therapies and beyond. J Clin Med. 2024;13:3098. 10.3390/jcm13113098 PMC11172489 38892809

[10] Pierzynowska K, Gaffke L, Cyske Z, Puchalski M, Rintz E, Bartkowski M, et al. Autophagy stimulation as a promising approach in treatment of neurodegenerative diseases. Metab Brain Dis. 2018;33:989–1008. 10.1007/s11011-018-0214-6 PMC6060747 29542037

[11] Ajoolabady A, Aslkhodapasandhokmabad H, Henninger N, Demillard LJ, Nikanfar M, Nourazarian A, et al. Targeting autophagy in neurodegenerative diseases: From molecular mechanisms to clinical therapeutics. Clin Exp Pharmacol Physiol. 2021;48:943–53. 10.1111/1440-1681.13500 PMC8204470 33752254

[12] Yang YP, Hu LF, Zheng HF, Mao CJ, Hu WD, Xiong KP, et al. Application and interpretation of current autophagy inhibitors and activators. Acta Pharmacol Sin. 2013;34:625–35. 10.1038/aps.2013.5 PMC4002883 23524572

[13] Pierzynowska K, Podlacha M, Gaffke L, Majkutewicz I, Mantej J, Węgrzyn A, et al. Autophagy-dependent mechanism of genistein-mediated elimination of behavioral and biochemical defects in the rat model of sporadic Alzheimer’s disease. Neuropharmacology. 2019;148:332–46. 10.1016/j.neuropharm.2019.01.030 30710571

[14] Pierzynowska K, Podlacha M, Gaffke L, Rintz E, Wiśniewska K, Cyske Z, et al. Correction of symptoms of Huntington disease by genistein through FOXO3-mediated autophagy stimulation. Autophagy. 2024;20:1159–82. 10.1080/15548627.2023.2286116 PMC11135876 37992314

[15] Sharifi-Rad J, Quispe C, Imran M, Rauf A, Nadeem M, Gondal TA, et al. Genistein: an integrative overview of its mode of action, pharmacological properties, and health benefits. Oxid Med Cell Longev. 2021;2021:3268136. 10.1155/2021/3268136 PMC8315847 34336089

[16] Rintz E, Gaffke L, Pierzynowska K, Podlacha M, Mantej J, Bednarek M, et al. Genistein – a natural antioxidant and its use in treatment of various diseases. In: Lewandrowski K-U, editor. Bentham Briefs in Biomedicine and Pharmacotherapy. Sharjah (UAE): Bentham Science; 2021. pp. 397–420.

[17] Guo TL, Chen Y, Xu HS, McDonough CM, Huang G. Gut microbiome in neuroendocrine and neuroimmune interactions: The case of genistein. Toxicol Appl Pharmacol. 2021;402:115130. 10.1016/j.taap.2020.115130 PMC7398836 32673657

[18] Yang R, Jia Q, Mehmood S, Ma S, Liu X. Genistein ameliorates inflammation and insulin resistance through mediation of gut microbiota composition in type 2 diabetic mice. Eur J Nutr. 2021;60:2155–68. 10.1007/s00394-020-02403-0 33068158

[19] Kesika P, Suganthy N, Sivamaruthi BS, Chaiyasut C. Role of gut-brain axis, gut microbial composition, and probiotic intervention in Alzheimer’s disease. Life Sci. 2021;264:118627. 10.1016/j.lfs.2020.118627 33169684

[20] Sharma VK, Singh TG, Garg N, Dhiman S, Gupta S, Rahman MH, et al. Dysbiosis and Alzheimer’s disease: A role for chronic stress? Biomolecules. 2021;11:678. 10.3390/biom11050678 PMC8147174 33946488

[21] Grünblatt E, Yde Ohki CM, Schmitt-Böhrer GA, Riederer P, Walitza S. Exploring the interplay of glucose metabolism, insulin resistance, and neurodegenerative pathologies: insights from streptozotocin and hypoglycaemic in vitro models. J Neural Transm. 2025;132:1479–91. 10.1007/s00702-025-02891-6 PMC12568790 39932549

[22] Oblak AL, Lin PB, Kotredes KP, Pandey RS, Garceau D, Williams HM, et al. Comprehensive evaluation of the 5XFAD mouse model for preclinical testing applications: A MODEL-AD study. Front Aging Neurosci. 2021;13:713726. 10.3389/fnagi.2021.713726 PMC8346252 34366832

[23] Van der Jeugd A, Blum D, Raison S, Eddarkaoui S, Buée L, D’Hooge R. Observations in THY-Tau22 mice that resemble behavioral and psychological signs and symptoms of dementia. Behav Brain Res. 2013;242:34–9. 10.1016/j.bbr.2012.12.008 23247080

[24] Pairojana T, Phasuk S, Suresh P, Huang SP, Pakaprot N, Chompoopong S, et al. Age and gender differences for the behavioral phenotypes of 3xTg Alzheimer’s disease mice. Brain Res. 2021;1762:147437. 10.1016/j.brainres.2021.147437 33753066

[25] Westi EW, Molhemi S, Hansen CT, Skoven CS, Knopper RW, Ahmad DA, et al. Comprehensive analysis of the 5xFAD mouse model of Alzheimer’s disease using dMRI, immunohistochemistry, and neuronal and glial functional metabolic mapping. Biomolecules. 2024;14(10):1294. 10.3390/biom14101294 PMC11505609 39456227

[26] Nonino F, Minozzi S, Sambati L, Del Giovane C, Baldin E, Bassi MC, et al. Amyloid-beta-targeting monoclonal antibodies for people with mild cognitive impairment or mild dementia due to Alzheimer’s disease. Cochrane Database Syst Rev. 2026;4:CD016297. 10.1002/14651858.CD016297 PMC13082890 41985900

[27] Pierzynowska K, Karaszewski B, Węgrzyn G. Genistein: a possible solution for the treatment of Alzheimer’s disease. Neural Regen Res. 2025;20:2903–05. 10.4103/NRR.NRR-D-24-00713 PMC11826469 39610096

[28] Kim KH, Dodsworth C, Paras A, Burton BK. High dose genistein aglycone therapy is safe in patients with mucopolysaccharidoses involving the central nervous system. Mol Genet Metab. 2013;109:382–5. 10.1016/j.ymgme.2013.06.012 23845234

[29] Lester E, Ooi FK, Bakkar N, Ayers J, Woerman AL, Wheeler J, et al. Tau aggregates are RNA-protein assemblies that mislocalize multiple nuclear speckle components. Neuron. 2021;109:1675–e919. 10.1016/j.neuron.2021.03.026 PMC8141031 33848474

[30] Smeralda W, Since M, Cardin J, Corvaisier S, Lecomte S, Cullin C, et al. β-Amyloid peptide interactions with biomimetic membranes: A multiparametric characterization. Int J Biol Macromol. 2021;181:769–77. 10.1016/j.ijbiomac.2021.03.107 33811932

[31] Banerjee S, Hashemi M, Zagorski K, Lyubchenko YL. Cholesterol in membranes facilitates aggregation of amyloid beta protein at physiologically relevant concentrations. ACS Chem Neurosci. 2021;12:506–16. 10.1021/acschemneuro.0c00688 33492944

[32] Hu KW, Fan HF, Lin HC, Huang JW, Chen YC, Shen CL, et al. Exploring the impact of glyoxal glycation on beta-amyloid peptide (Abeta) aggregation in Alzheimer’s disease. J Phys Chem B. 2021;125:5559–71. 10.1021/acs.jpcb.1c02797 34019761

[33] Le W, editor. Autophagy: Biology and Diseases. Springer, Singapore. 2020. ISBN: 978-981-15-4271-8.

[34] Bonet-Costa V, Herranz-Pérez V, Blanco-Gandía M, Mas-Bargues C, Inglés M, Garcia-Tarraga P, et al. Clearing amyloid-β through PPARγ/ApoE activation by genistein is a treatment of experimental Alzheimer’s disease. J Alzheimers Dis. 2016;51:701–11. 10.3233/JAD-151020 26890773

[35] Park YJ, Ko JW, Jeon S, Kwon YH. Protective effect of genistein against neuronal degeneration in *ApoE*^-/-^ mice fed a high-fat diet. Nutrients. 2016;8:692. 10.3390/nu8110692 PMC5133079 27809235

[36] Wang Y, Cai B, Shao J, Wang TT, Cai RZ, Ma CJ, et al. Genistein suppresses the mitochondrial apoptotic pathway in hippocampal neurons in rats with Alzheimer’s disease. Neural Regen Res. 2016;11:1153–8. 10.4103/1673-5374.187056 PMC4994461 27630702

[37] Zhou X, Yuan L, Zhao X, Hou C, Ma W, Yu H, et al. Genistein antagonizes inflammatory damage induced by β-amyloid peptide in microglia through TLR4 and NF-κB. Nutrition. 2014;30:90–5. 10.1016/j.nut.2013.06.006 24290604

[38] Bagheri M, Joghataei MT, Mohseni S, Roghani M. Genistein ameliorates learning and memory deficits in amyloid β(1–40) rat model of Alzheimer’s disease. Neurobiol Learn Mem. 2011;95:270–6. 10.1016/j.nlm.2010.12.001 21144907

[39] Nagu P, Parashar A, Behl T, Mehta V. Gut microbiota composition and epigenetic molecular changes connected to the pathogenesis of Alzheimer’s disease. J Mol Neurosci. 2021;71:1436–55. 10.1007/s12031-021-01829-3 33829390

[40] Dey P. Targeting gut barrier dysfunction with phytotherapies: Effective strategy against chronic diseases. Pharmacol Res. 2020;161:105135. 10.1016/j.phrs.2020.105135 32814166

[41] Snow AD, Cummings JA, Lake T. The unifying hypothesis of Alzheimer’s disease: Heparan sulfate proteoglycans/glycosaminoglycans are key as first hypothesized over 30 years ago. Front Aging Neurosci. 2021;13:710683. 10.3389/fnagi.2021.710683 PMC8521200 34671250

[42] Tanjak P, Thiantanawat A, Watcharasit P, Satayavivad J. Genistein reduces the activation of AKT and EGFR, and the production of IL6 in cholangiocarcinoma cells involving estrogen and estrogen receptors. Int J Oncol. 2018;53:177–88. 10.3892/ijo.2018.4375 29693152

[43] Moskot M, Jakóbkiewicz-Banecka J, Kloska A, Smolińska E, Mozolewski P, Malinowska M, et al. Modulation of expression of genes involved in glycosaminoglycan metabolism and lysosome biogenesis by flavonoids. Sci Rep. 2015;5:9378. 10.1038/srep09378 PMC4369724 25797591

[44] Jakóbkiewicz-Banecka J, Piotrowska E, Narajczyk M, Barańska S, Wegrzyn G. Genistein-mediated inhibition of glycosaminoglycan synthesis, which corrects storage in cells of patients suffering from mucopolysaccharidoses, acts by influencing an epidermal growth factor-dependent pathway. J Biomed Sci. 2009;16:26. 10.1186/1423-0127-16-26 PMC2653532 19272193

[45] Pierzynowska K, Gaffke L, Cyske Z, Jerzemowska G, Węgrzyn G, Podlacha M. Potential effects of genistein in preventing progressive loss of cognitive functions in wild-type mice. Nutr Neurosci. 2026;29:1153–73. 10.1080/1028415X.2026.2634117 41784650

[46] León-Arcia K, Andrade-Guerrero J, Martínez-Orozco H, Villegas-Rojas MM, Pérez-Segura I, Ramírez IL, et al. First unified time-course of Alzheimer’s-like pathology in the intracerebroventricular streptozotocin-rat model: A systematic review. Ageing Res Rev. 2026;113:102918. 10.1016/j.arr.2025.102918 41093165

[47] Pierzynowska K, Kamińska T, Węgrzyn G. One drug to treat many diseases: unlocking the economic trap of rare diseases. Metab Brain Dis. 2020;35:1237–40. 10.1007/s11011-020-00617-z PMC7584527 32926291

