# Supplementary material for: The use of three murine genetic models of Alzheimer’s disease reveals efficacy of 4′,5,7-trihydroxyisoflavone in alleviating cognitive deficits and reducing levels of amyloid-beta and hyperphosphorylated tau protein
Source: Lab Anim Res. 2026 Oct 22;42:41. doi: 10.1186/s42826-026-00302-9 (PMC13688988; doi:10.1186/s42826-026-00302-9)
Supplement: Supplementary file 1 [file 42826_2026_302_MOESM1_ESM.pdf]

SUPPLEMENTARY MATERIAL

Table S1. Details of statistical analyses of results presented in Figures 2-7.

Figure	Panel	Sex	Statistical test	Interaction	Time	Group	Post hoc
Fig. 2	A	Male	Ordinary two-way ANOVA	F(15,168)=10.76, *p*<0.0001	F(3,168)=13.38, *p*<0.0001	F(5,168)=93.82, *p*<0.0001	Tukey
Fig. 2	A	Female	Ordinary two-way ANOVA	F(15,168)=13.86, *p*<0.0001	F(3,168)=33.47, *p*<0.0001	F(5,168)=114.2, *p*<0.0001	Tukey
Fig. 2	B	Male	Ordinary two-way ANOVA	F(15,168)=11.92, *p*<0.0001	F(3,168)=15.39, *p*<0.0001	F(5,168)=102.5, *p*<0.0001	Tukey
Fig. 2	B	Female	Ordinary two-way ANOVA	F(15,168)=12.38, *p*<0.0001	F(3,168)=28.32, *p*<0.0001	F(5,168)=98.22, *p*<0.0001	Tukey
Fig. 2	C	Male	Ordinary two-way ANOVA	F(15,168)=11.78, *p*<0.0001	F(3,168)=17.55, *p*<0.0001	F(5,168)=102.1, *p*<0.0001	Tukey
Fig. 2	C	Female	Ordinary two-way ANOVA	F(15,168)=13.18, *p*<0.0001	F(3,168)=39.55, *p*<0.0001	F(5,168)=114.0, *p*<0.0001	Tukey
Fig. 3	A	Male	Ordinary two-way ANOVA	F(15,168)=1.074, *p*=0.3845	F(3,168)=35.80, *p*<0.0001	F(5,168)=17.67, *p*<0.0001	Tukey
Fig. 3	A	Female	Ordinary two-way ANOVA	F(15,168)=0.6647, *p*=0.8162	F(3,168)=63.77, *p*<0.0001	F(5,168)=4.018, *p*=0.0018	Tukey
Fig. 3	B	Male	Ordinary two-way ANOVA	F(15,168)=1.722, *p*=0.0507	F(3,168)=112.8, *p*<0.0001	F(5,168)=25.75, *p*<0.0001	Tukey
Fig. 3	B	Female	Ordinary two-way ANOVA	F(15,168)=0.6519, *p*=0.8280	F(3,168)=115.1, *p*<0.0001	F(5,168)=12.15, *p*<0.0001	Tukey

Figure	Panel	Sex	Statistical test	Interaction	Time	Group	Post hoc
Fig. 3	C	Male	Ordinary two-way ANOVA	$F(15,168)=1.316$, $p=0.1973$	$F(3,168)=110.3$, $p<0.0001$	$F(5,168)=22.18$, $p<0.0001$	Tukey
Fig. 3	C	Female	Ordinary two-way ANOVA	$F(15,168)=0.8481$, $p=0.6231$	$F(3,168)=109.1$, $p<0.0001$	$F(5,168)=12.42$, $p<0.0001$	Tukey
Fig. 4	A	Male	Ordinary two-way ANOVA	$F(10,126)=5.590$, $p<0.0001$	$F(2,126)=11.09$, $p<0.0001$	$F(5,126)=851.7$, $p<0.0001$	Tukey
Fig. 4	A	Female	Ordinary two-way ANOVA	$F(10,126)=3.191$, $p=0.0011$	$F(2,126)=9.517$, $p=0.0001$	$F(5,126)=677.2$, $p<0.0001$	Tukey
Fig. 4	B	Male	Ordinary two-way ANOVA	$F(10,126)=5.411$, $p<0.0001$	$F(2,126)=10.73$, $p<0.0001$	$F(5,126)=881.9$, $p<0.0001$	Tukey
Fig. 4	B	Female	Ordinary two-way ANOVA	$F(10,126)=3.161$, $p=0.0012$	$F(2,126)=9.474$, $p=0.0001$	$F(5,126)=736.1$, $p<0.0001$	Tukey
Fig. 4	C	Male	Ordinary two-way ANOVA	$F(10,126)=4.890$, $p<0.0001$	$F(2,126)=11.10$, $p<0.0001$	$F(5,126)=943.0$, $p<0.0001$	Tukey
Fig. 4	C	Female	Ordinary two-way ANOVA	$F(10,126)=3.044$, $p=0.0017$	$F(2,126)=9.241$, $p=0.0002$	$F(5,126)=781.6$, $p<0.0001$	Tukey
Fig. 5	A	Male	Ordinary two-way ANOVA	$F(10,126)=14.39$, $p<0.0001$	$F(2,126)=39.15$, $p<0.0001$	$F(5,126)=410.8$, $p<0.0001$	Tukey
Fig. 5	A	Female	Ordinary two-way ANOVA	$F(10,126)=50.64$, $p<0.0001$	$F(2,126)=97.42$, $p<0.0001$	$F(5,126)=521.4$, $p<0.0001$	Tukey

Figure	Panel	Sex	Statistical test	Interaction	Time	Group	Post hoc
Fig. 5	B	Male	Ordinary two-way ANOVA	$F(10,126)=9.236$, $p<0.0001$	$F(2,126)=9.990$, $p<0.0001$	$F(5,126)=225.1$, $p<0.0001$	Tukey
Fig. 5	B	Female	Ordinary two-way ANOVA	$F(10,126)=3.007$, $p=0.0019$	$F(2,126)=2.735$, $p=0.0688$	$F(5,126)=312.1$, $p<0.0001$	Tukey
Fig. 6	A (Aβ40)	Male	Ordinary two-way ANOVA	$F(10,126)=9.795$, $p<0.0001$	$F(2,126)=12.48$, $p<0.0001$	$F(5,126)=309.8$, $p<0.0001$	Tukey
Fig. 6	A (Aβ40)	Female	Ordinary two-way ANOVA	$F(10,126)=8.727$, $p<0.0001$	$F(2,126)=19.76$, $p<0.0001$	$F(5,126)=381.0$, $p<0.0001$	Tukey
Fig. 6	A (Aβ42)	Male	Ordinary two-way ANOVA	$F(10,126)=122.5$, $p<0.0001$	$F(2,126)=207.3$, $p<0.0001$	$F(5,126)=868.6$, $p<0.0001$	Tukey
Fig. 6	A (Aβ42)	Female	Ordinary two-way ANOVA	$F(10,126)=137.6$, $p<0.0001$	$F(2,126)=201.8$, $p<0.0001$	$F(5,126)=1352$, $p<0.0001$	Tukey
Fig. 6	B (Aβ40)	Male	Ordinary two-way ANOVA	$F(10,126)=9.751$, $p<0.0001$	$F(2,126)=12.54$, $p<0.0001$	$F(5,126)=264.2$, $p<0.0001$	Tukey
Fig. 6	B (Aβ40)	Female	Ordinary two-way ANOVA	$F(10,126)=8.727$, $p<0.0001$	$F(2,126)=19.76$, $p<0.0001$	$F(5,126)=327.3$, $p<0.0001$	Tukey
Fig. 6	B (Aβ42)	Male	Ordinary two-way ANOVA	$F(10,126)=135.4$, $p<0.0001$	$F(2,126)=242.3$, $p<0.0001$	$F(5,126)=740.8$, $p<0.0001$	Tukey
Fig. 6	B (Aβ42)	Female	Ordinary two-way ANOVA	$F(10,126)=146.8$, $p<0.0001$	$F(2,126)=221.1$, $p<0.0001$	$F(5,126)=1123$, $p<0.0001$	Tukey

Figure	Panel	Sex	Statistical test	Interaction	Time	Group	Post hoc
Fig. 7	A	Male	Ordinary two-way ANOVA	$F(10,126)=73.13$, $p<0.0001$	$F(2,126)=131.4$, $p<0.0001$	$F(5,126)=1179$, $p<0.0001$	Tukey
Fig. 7	A	Female	Ordinary two-way ANOVA	$F(10,126)=95.31$, $p<0.0001$	$F(2,126)=180.6$, $p<0.0001$	$F(5,126)=1095$, $p<0.0001$	Tukey
Fig. 7	B	Male	Ordinary two-way ANOVA	$F(10,126)=64.50$, $p<0.0001$	$F(2,126)=126.9$, $p<0.0001$	$F(5,126)=718.4$, $p<0.0001$	Tukey
Fig. 7	B	Female	Ordinary two-way ANOVA	$F(10,126)=100.4$, $p<0.0001$	$F(2,126)=210.3$, $p<0.0001$	$F(5,126)=866.9$, $p<0.0001$	Tukey
